# Cov-caldas: A new COVID-19 chest X-Ray dataset from state of Caldas-Colombia

**DOI:** 10.1038/s41597-022-01576-z

**Published:** 2022-12-07

**Authors:** Jesús Alejandro Alzate-Grisales, Alejandro Mora-Rubio, Harold Brayan Arteaga-Arteaga, Mario Alejandro Bravo-Ortiz, Daniel Arias-Garzón, Luis Humberto López-Murillo, Esteban Mercado-Ruiz, Juan Pablo Villa-Pulgarin, Oscar Cardona-Morales, Simon Orozco-Arias, Felipe Buitrago-Carmona, Maria Jose Palancares-Sosa, Fernanda Martínez-Rodríguez, Sonia H. Contreras-Ortiz, Jose Manuel Saborit-Torres, Joaquim Ángel Montell Serrano, María Mónica Ramirez-Sánchez, Mario Alfonso Sierra-Gaber, Oscar Jaramillo-Robledo, Maria de la Iglesia-Vayá, Reinel Tabares-Soto

**Affiliations:** 1 Department of Electronics and Automation, Universidad Autónoma de Manizales, Manizales, 170001 Colombia; 2 Department of Computer Science, Universidad Autónoma de Manizales, Manizales, 170001 Colombia; 3 Department of Systems and Informatics, Universidad de Caldas, Manizales, 170004 Colombia; 4 Biotechnology Interdisciplinar Professional Unit, Instituto Politécnico Nacional, Ciudad de México, 07300 México; 5 Department of Translational Bioengineering, Universidad de Guadalajara, Guadalajara, 44430 México; 6 School of Engineering, Universidad Tecnológica de Bolívar, Cartagena de Indias, 130001 Colombia; 7 Unidad Mixta de Imagen Biomédica FISABIO-CIPF. Fundación para el Fomento de la Investigación Sanitario y Biomédica de la Comunidad Valenciana, Valencia, 46020 Spain; 8 Unidad Imágenes Diagnósticas, S.E.S Hospital Universitario de Caldas, Manizales, 170004 Colombia

**Keywords:** Radiography, Pathology

## Abstract

The emergence of COVID-19 as a global pandemic forced researchers worldwide in various disciplines to investigate and propose efficient strategies and/or technologies to prevent COVID-19 from further spreading. One of the main challenges to be overcome is the fast and efficient detection of COVID-19 using deep learning approaches and medical images such as Chest Computed Tomography (CT) and Chest X-ray images. In order to contribute to this challenge, a new dataset was collected in collaboration with “S.E.S Hospital Universitario de Caldas” (https://hospitaldecaldas.com/) from Colombia and organized following the Medical Imaging Data Structure (MIDS) format. The dataset contains 7,307 chest X-ray images divided into 3,077 and 4,230 COVID-19 positive and negative images. Images were subjected to a selection and anonymization process to allow the scientific community to use them freely. Finally, different convolutional neural networks were used to perform technical validation. This dataset contributes to the scientific community by tackling significant limitations regarding data quality and availability for the detection of COVID-19.

## Abstract (summary)

**Table:** 

Measurement(s)	Radiologic Examination
Technology Type(s)	Artificial Intelligence
Sample Characteristic - Location	Colombia

## Background & Summary

Since the outbreak of COVID-19 in late 2019, and after being declared by the World Health Organization (WHO) as a pandemic in March 2020, the research community in Artificial Intelligence (AI) has concentrated its efforts in developing tools to aid disease diagnosis in order to control, both effectively and efficiently, its spreading. These tools, most of which are based on Machine Learning (ML) or Deep Learning (DL) models, aim to overcome the limitations of conventional laboratory tests, such as the Polymerase Chain Reaction (PCR)^1^. Some of these limitations include: (i) the extended period between the sample collection and test result, (ii) the low availability and (iii) high cost, especially in developing countries.

Chest Computed Tomography (CT) and X-Ray images have been the primary source of information to develop the classification models since they are used by radiologists to detect the disease and estimate its severity based on the presence and characteristics of affected regions in the lungs known as Ground Glass Opacities. In general, both CT and X-Ray are medical imaging techniques that provide images of the internal structure of the human body using radiation. With these techniques, it is possible to capture and differentiate bones, soft tissues, fat tissue, and gas areas based on the color they appear on the image^2^. The CT produces higher quality images^3^ in 3D as opposed to the 2D images generated by the X-Ray technique, but at the expense of a much higher radiation dose^4^, which is harmful to patients in the long term, and requires more complex equipment that is less accessible than a conventional X-Ray device. Based on these characteristics, researchers, working on the development of ML or DL models for disease detection, have turned their attention to Chest X-Ray images.

Data quality and availability are critical factors when developing systems based on DL models, especially in the health sector where the generalization ability of the model is crucial for success in a real-world situation. Additionally, DL models require the images to be traceable in terms of the source and acquisition conditions to avoid inducing bias to the model based on factors outside image content. In this sense, it is necessary to identify the published datasets that are used to train and evaluate models for COVID-19 disease detection. In general, the images for positive cases are taken from six primary sources, these are: IEEE8023^5^, BIMCV-COVID19+^6^, Cancer Image Archive^7^, ML Hannover^8^, BRIXIA^9^, and HM Hospitales^10^. The main limitation with these datasets is the low quantity of images, some lower than others, which leads researchers to join multiple datasets generating data distribution issues in multiple cases. On the other hand, for the images in the negative class researchers have used mainly three types of images: from healthy patients, from patients with pneumonia non-associated to COVID-19, and images of different pathologies. The following five sources are the most used for negative class images: Padchest^11^, BIMCV-COVID-^12^, CheXpert^13^, RSNA^14^, Chest X-Ray Images (Pneumonia)^15^.

In order to contribute to the development of DL-based detection models, this paper presents a novel dataset collected in collaboration with “S.E.S Hospital Universitario de Caldas”, a healthcare institution located in the city of Manizales, Colombia. This dataset consists of 3,077 Chest X-Ray images positive for COVID-19 from studies involving 657 subjects, and 4,230 images negative for COVID-19 from 2,164 different subjects. Furthermore, COVID-19 detection models based on Convolutional Neural Networks (CNNs) were trained and evaluated by using the presented dataset, aiming to establish a set of benchmark detection performances.

## Methods

### Design considerations

In the dataset development of medical images, data must be completely structured to facilitate its manipulation. Besides, the non-identifiability of the patient must be guaranteed and the data must be curated to avoid biases in the training of the AI models. The images were collected under the approval, and supervision, of the “S.E.S Hospital Universitario de Caldas” ethics committee, and every patient gives their consent to the use of properly anonymized data upon entry to the healthcare institution. Therefore, the data curation follow the next steps:

- Data acquisition: raw data capture from hospitals, clinics or health care providers.
- De-identification: Removal or pseudonymization of the patient identification data.
- Data transfer: Anonymized or pseudonymized data are transferred to a secure storage site.
- Data curation: Procedure performed by radiologists and segmentation specialists to obtain a correct labeling of the data.
- User access: Repository design for free access to users for any purpose.

A dataset of medical images for AI applications must guarantee at least the use of pseudonyms and, if possible, the complete anonymization of patient data, since medical images contain information that easily allows the identification of patients’ non-transferable personal data, such as name, identification, date and time of the x-ray session. This medical information and its security is protected by the legislation itself and is associated with strict ethical standards. HIPAA (Health Insurance Portability and Accountability Act) proposes a set of best practices to protect the confidentiality, integrity and availability of information. In particular, it addresses the unique needs of information security management in the healthcare sector and its operating environments.

Medical imaging is guided by the DICOM (Digital Imaging and Communications in Medicine) standard, which aims to ensure interoperability between heterogeneous medical imaging equipment and systems. This standard fully controls the handling of image-related information, including the rules for transferring these images securely when using ISO-OSI and TCP/IP (Open System Interconnection and Transmission Control Protocol/Internet Protocol, respectively) communication protocols.

The data in DICOM format that come from health care providers are data without a clear structure and difficult to work with since they have no order or labeling that allows their implementation in AI projects. To solve this problem, FISABIO^16^ has defined and proposed a methodology to standardize the organization and management of medical image data: The Medical Imaging Data Structure (MIDS). MIDS provides the basis for the dataset approach as an attempt to facilitate the generation of large datasets for AI research, and corresponds to a BIDS extension Proposal 25 (BEP025) found at https://bids.neuroimaging.io/get_involved.html#extending-the-bids-specification. This approach will enable pioneering progress in the development of ready-to-use datasets for use in AI projects, as well as the labeling and annotation of these datasets.

### Collection and anonymization

**Fig. 1:**
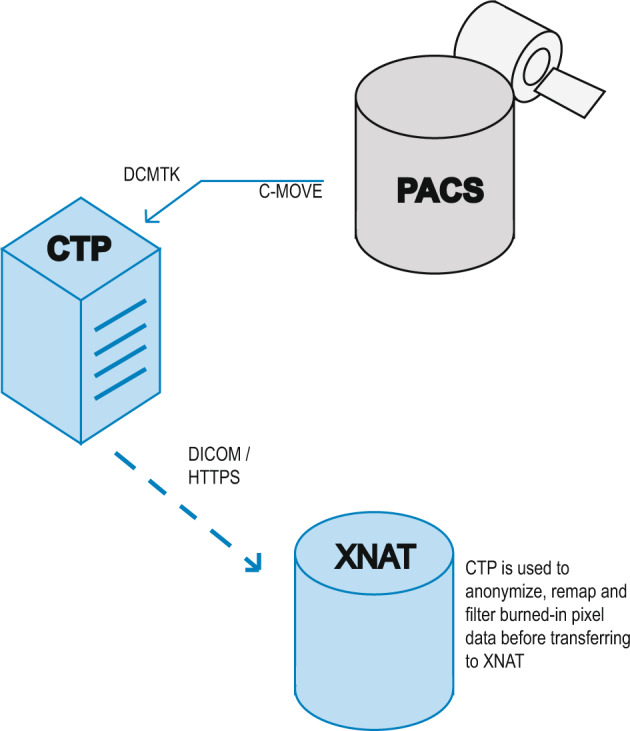
Information flow from the origin where the data is identified in the PACS of the health institution to the final storage of the raw data in the PACS of the health institution to the final storage of the raw data in XNAT.

The images collected by specialized health centers contain information that must be processed to protect the identity of the patients. For this reason, a process must be established to guarantee the security of sensitive data from the images acquisition to their final manipulation by researchers. The Fig. 1 illustrates concisely how the data is processed to be later released and freely manipulated by other researchers.

PACS (Picture Archiving and Communication System) is a file containing the X-ray images of each patient captured by healthcare centers stored in DICOM format^17^. For this purpose, the following confidentiality profiles are established as factors to be taken into account when processing data in DICOM format.

- Delete the basic profile (patient’s name, physician’s name, etc.)
- Keep private information secure.
- Keep the UID (identifier in the data list does not identify the patient).
- Maintain device identity.
- Maintain patient characteristics.
- Preserve complete dates (Preserving dates is considered a risk because it limits the set of patients to which an image may belong. However, in some cases, retaining dates may be necessary for research purposes).
- Modify dates while preserving the space of time between each date.
- Clean up descriptors (patient name may be present in demographic or physical descriptors).
- Clean structured content
- Clean graphics

The GitHub repository https://github.com/BIMCV-CSUSP/Smart-Upload was implemented. It contains a set of scripts to upload DICOM files from a neutral or PACS file to the XNAT (https://www.xnat.org/) platform through the CTP (Clinical Trial Processor) server (https://www.rsna.org/research/imaging-research-tools). This script will clean and decompress the images before uploading them to the CTP. In addition, the CTP will anonymize and upload the files to the XNAT platform.

Once the images have been anonymized and uploaded to XNAT they will be accessible to anyone interested in researching with this type of data, which facilitates the manipulation of the data for researchers in data science, data analysis, artificial intelligence, and other projects. At this stage we have completely anonymized data, as all data that could link a patient or health personnel to an x-ray image has been removed.

## Data Records

The proposed dataset is publicly available and can be downloaded from Figshare^18^. The dataset was collected in collaboration with “S.E.S Hospital Universitario de Caldas” (Manizales, Caldas, Colombia). It contains 7,307 chest X-ray images, and divided into 3,077 COVID-19 positive and 4,230 COVID-19 negative images.

**Fig. 2:**
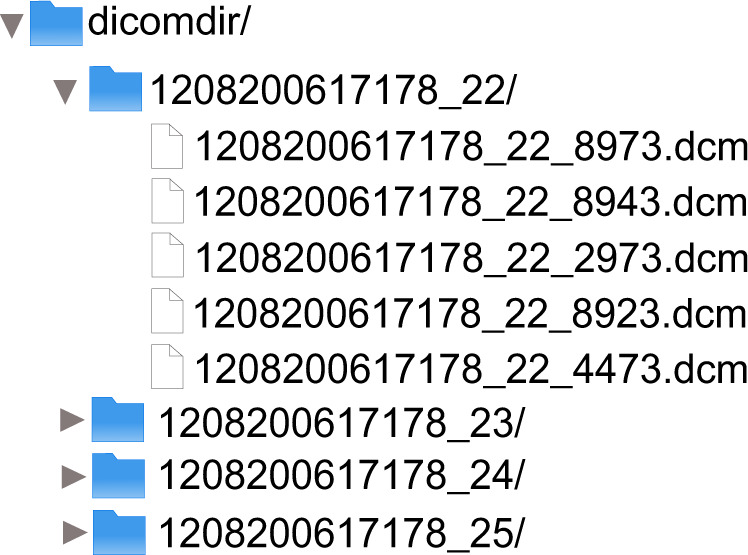
Data structure in DICOM format.

Likewise, for the COVID-19 negative and COVID-19 positive sets, there are three ways of structuring the data. The first way consists of images downloaded directly from XNAT after being anonymized; the format of these images is DICOM, and contains the metadata of these images that did not require anonymization. The structure of this modality can be seen in Fig. 2. In this case there is a root folder (*dicomdir*) and a separate folder for each patient that contains all the performed studies.

**Fig. 3:**
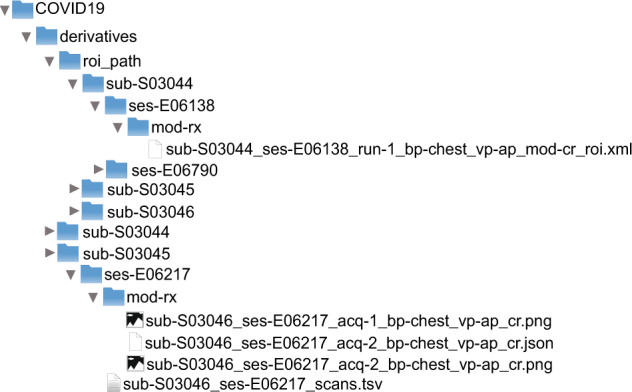
Data structure in MIDS format.

Correspondingly, as can be seen in Fig. 3, the second modality corresponds to images organized according to the MIDS structure. In this structure, there is the main folder of the project. Inside it is the folder derivatives, which contains the roi_path folder and will have the XML files of each session of each of the subjects; these files correspond to the coordinates of the segmentation maps if they have been generated in the XNAT; otherwise, these files will not exist. Likewise, there are folders for each subject of the study with their corresponding sessions and tables with the description of the dates of these sessions. Alike, within each session, the radiographic image is associated in png format with the same name as a JSON file with the metadata extracted from the DICOM format.

Finally, to perform the technical validations using deep learning techniques, the images have been separated into training, validation, and test subsets, using three different random state seeds and maintaining a 70-15-15 percentages distribution in the number of images in each of the subsets. This structuring modality has the images packed in numpy array files.

## Technical Validation

**Fig. 4:**
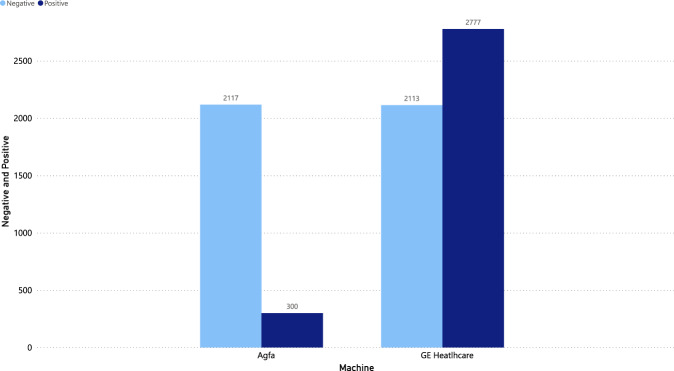
Distribution of X-ray equipment brands.

All images were taken with two brands of X-ray equipment, GE Healthcare model Optima XR646 and AGFA model CR85. As Fig. 4 shows, the equipment brand with the most images taken is GE Healthcare with 2,777 COVID-19 positive and 2,113 COVID-19 negative images. Likewise, images taken with AGFA brand equipment show 300 and 2,117 images for COVID-19 positive and COVID-19 negative, respectively.

### COVID-19 positive

**Fig. 5:**
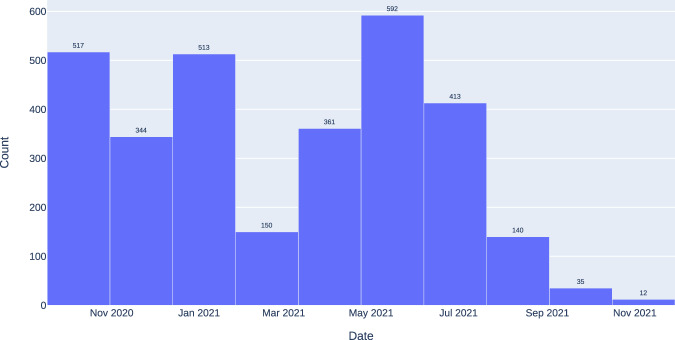
Study date distribution of COVID-19 positive images.

For the positive cases of COVID-19, 3,077 chest X-ray images were collected from studies performed on 657 subjects between September 17, 2020, and November 29, 2021. Figure 5 shows the distribution of the study date of each image, where the most significant number of images presented in the dataset belongs to studies performed between March and August 2021. Similarly, the images have different sizes. For instance, the smallest image in this dataset contains 646 × 835 pixels, while the largest is 2,828 × 2,320 pixels. The labeling process for the positive COVID-19 images was based on a positive result in a conventional laboratory test, such as the PCR.

**Fig. 6:**
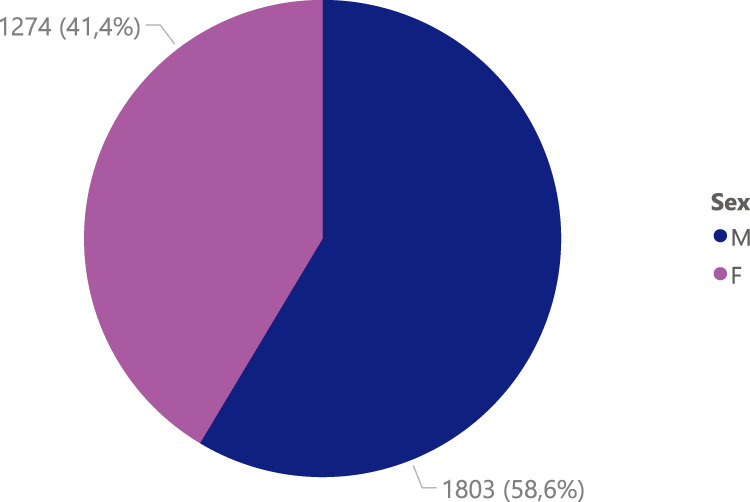
Gender distribution of COVID-19 positive images.

**Fig. 7:**
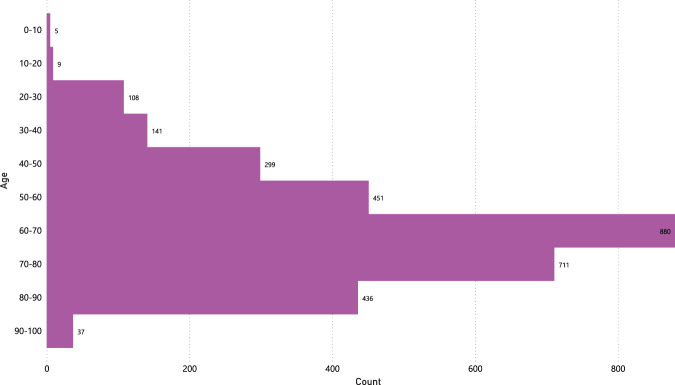
Age distribution of COVID-19 positive images.

Also, there is a different distribution between the gender of the subjects, where the number of male subjects is 1,803 and female subjects is 1,274, as shown in Fig. 6. Furthermore, Fig. 7 shows the age distribution of the COVID-19 positive subjects who underwent radiography, and it can be evidenced how most of the subjects are between 50 and 80 years old.

### COVID-19 negative

**Fig. 8:**
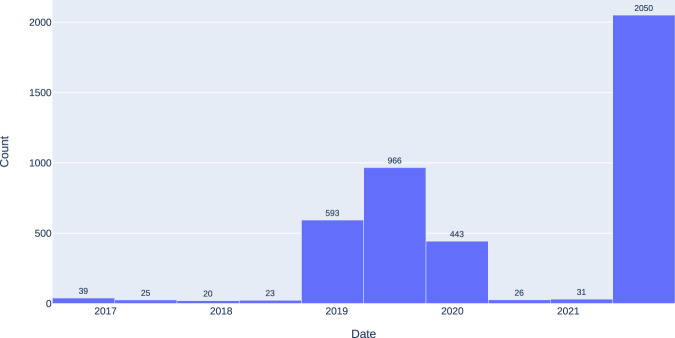
Study date distribution of COVID-19 negative images.

Analogously, the negative cases for COVID-19 corresponds to 4,230 images from 2,164 subjects between July 17, 2016, and April 12, 2021. Figure 8 shows the distribution of the study date of each image, where an essential part of the images are collected before the first case of COVID-19 was detected in Colombia on March 6, 2020^19^. Furthermore, the other part of the images, corresponding to the dates on which positive COVID-19 cases were detected, belongs to subjects in whom the test had a negative result. Similarly to the images of the positive cases of COVID-19, these images have different sizes, where the smaller image contains 1,128 × 1,692 pixels and the larger one 2,970 × 2,460 pixels. The negative class label is ensured by the date of the study for those performed before 2020, and based on a negative PCR result for studies from 2020 and 2021.

**Fig. 9:**
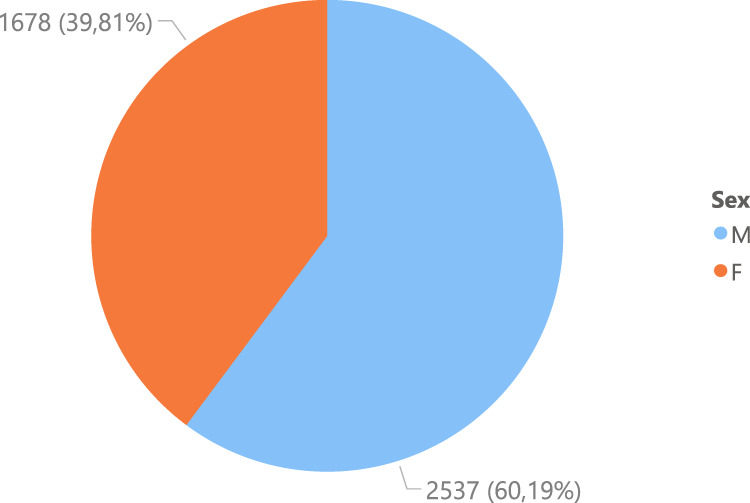
Gender distribution of COVID-19 negative images.

**Fig. 10:**
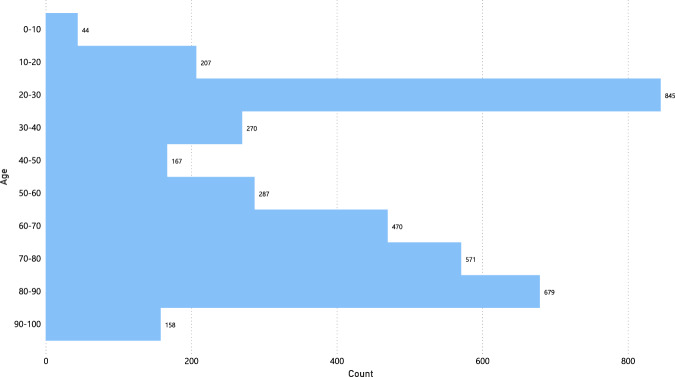
Age distribution of COVID-19 negative images.

Furthermore, the gender distribution of the negative images is similar to the positive images. The majority of the images correspond to male subjects with 2537 images and the smaller portion to female subjects with 1678 images, as shown in Fig. 9. Finally, Fig. 10 shows the age distribution of the subjects, where a large part corresponds to young patients between 20 and 30 years old. However, there is also an essential part of subjects between 60 and 90 years old, similar to COVID-19 positive part in Fig. 7.

### Experiments

With the dataset presented in this paper, we developed and tested a set of deep learning experiments. One of the most critical aspects of the development of the experiments consists in ensuring that COVID-19 is really classified. To this end, the presented experiments took into account the same conditions of the X-ray-taking equipment. Only the images generated with the GE Healthcare X-ray equipment were taken from the proposed dataset since this has most of the images for positive and negative COVID-19, as shown in Fig. 4. With this, we ensure that COVID-19 is classified and not a type of image given the capture conditions of the equipment. This experiments show the deep learning performance with different data distributions (seeds). The data distributions are verified so that the training, validation, and test datasets have different patients. This verification prevents that the CNN only recognize a specific patient. In the same way, models were applied with 2 distributions of the dataset, the first one consisting of the complete images, and for the second one a segmentation of the lung was performed, following the architecture and configuration proposed in^20^. In both distributions normalization was applied as part of the preprocessing.

#### Lung segmentation

There are multiple ways to perform image segmentation, in this case we implemented the Deep Learning model Unet^21^, which has been shown to achieve good performance in segmentation of medical images. During training, this model receives a Chest X-Ray image as input and a mask with ones on the reconstruction area and zeros in the rest as the target; at test time, model input is a Chest X-Ray image, and the output is the predicted mask.

Unet models are often defined in terms of the number of filters in the contraction blocks (See Eq. 1), where *F*_0_ is the number of the filters on the first block, and *i* corresponds to the index of the block. Eq. 2 shows the number of filters on each expansion block where *F*_*f*_ is the number of filters on the last contraction block. The difference between the contraction and the expansion blocks is that the latter uses transposed convolutions instead of the regular convolutions used in the contraction stage. In our experiment *F*_0_ was set experimentally to 64.

1 $$\#Filters={F}_{0}\ast {2}^{i-1}$$

2 $$\#Filters=\frac{{F}_{f}}{{2}^{i}}$$

Kernel size in convolutional layers was set to 3 × 3, using he-normal for kernel initialization, and padding same. For the Maxpooling layers, the pool size was 2 × 2. The Dropout rate in the first blocks of each stage was 0.1, 0.2 for the third and fourth blocks, and 0.3 for the last block. The kernel size was reduced to 2 × 2 on the transposed convolutional layers, with a stride of 2 × 2, and padding same. Finally, the last convolutional layer uses one filter of kernel size 1 × 1.

#### Data distributions for the experiments

- Seed 1:
  - Train, 3,213: negatives, 1,389; positives, 1,824
  - Test, 701: negatives, 303; positives, 398
- Seed 2:
  - Train, 3,237: negatives, 1,409; positives, 1,828
  - Test, 685: negatives, 299; positives, 386
- Seed 3:

- Train, 3,211: negatives, 1,389; positives, 1,822

- Test, 730: negatives, 300; positives, 430

#### Results

Image classification problems have been solved using CNNs. In this work, different CNN-based models are present in the Keras library, which includes MobilNet^22^, Xception^23^, EfficieNet^24^, VGG16^25^, VGG19^25^, InceptionResNetV2^26^, InceptionV3^26^, DenseNet201^27^, ResNet152V2^28^, ResNet50^29^, among others, were used, where all models were tested, and the three best models were chosen with each seed. With each of these models, transfer learning was used to improve the training resource requirements and the accuracy and convergence. Model weights were obtained by training on ImageNet, a dataset with millions of images and 1000 possible labels^30^. Four dense layers were used for classification, using 1024 neurons in the first dense layer, 512 in the second layer, 64 in the third, and 2 in the fourth dense layer. The activation function used for the first three dense layers is ReLu and Softmax for the fourth dense layer. Adam optimizer with a learning rate of 0.0001 and Categorical Crossentropy is used.

Tables 1, 2 show the experimental results with the test data and each partition seed. The results achieved in the different experiments proposed are encouraging, with accuracies about 87% and 84% for the best models, especially when taking into account the homogeneity in the acquisition conditions on the images for both classes, using the same radiology equipment and a similar distribution of gender and age, which leads to thinking of a true detection of COVID-19 disease. It is worth mentioning, that these results may be valuable in terms of establishing a benchmark that allows comparison with future experiments.

**Table 1:** Networks performance on Colombian test dataset without lung segmentation.

CNN architecture	Accuracy	Specificity	F1 score	Recall	Precision	FN	FP	TN	TP
*Seed 1*									
InceptionV3	**87.73**	90.10	88.83	85.93	91.94	56	30	273	342
VGG16	86.31	**92.08**	87.17	81.91	**93.14**	72	**24**	**279**	326
ResNet152V2	85.88	84.16	87.52	**87.19**	87.85	**51**	48	255	**347**
*Seed 2*									
VGG16	**87.15**	84.95	**88.63**	88.86	88.40	43	45	254	343
InceptionV3	86.28	**86.62**	87.60	86.01	**89.25**	54	**40**	**259**	332
ResNet152V2	86.13	81.94	87.90	**89.38**	86.47	**41**	54	245	345
*Seed 3*									
ResNet152V2	**86.16**	84.00	**88.19**	**87.67**	88.71	**53**	48	252	**377**
DenseNet201	84.66	80.33	87.07	**87.67**	86.47	**53**	59	241	377
InceptionV3	84.52	**87.33**	86.27	82.56	**90.33**	75	**38**	**262**	355

**Table 2:** Networks performance on Colombian test dataset with lung segmentation.

CNN architecture	Accuracy	Specificity	F1 score	Recall	Precision	FN	FP	TN	TP
*Seed 1*									
InceptionV3	**84.88**	**82.50**	**86.68**	86.68	**86.68**	53	**53**	**250**	345
VGG16	84.59	80.20	86.63	87.94	85.37	48	60	243	350
DenseNet201	84.31	77.56	86.62	**89.45**	83.96	**42**	68	235	**356**
*Seed 2*									
InceptionResNetV2	**84.96**	83.28	**86.61**	**86.27**	86.95	**53**	50	249	**333**
DenseNet201	84.09	81.27	85.94	**86.27**	85.60	**53**	56	243	**333**
InceptionV3	83.36	**84.28**	84.84	82.64	**87.16**	67	47	**252**	319
*Seed 3*									
VGG16	**83.70**	79.00	**86.27**	**86.98**	85.58	**56**	63	237	**374**
InceptionResNetV2	82.60	80.67	85.04	83.95	86.16	69	58	242	361
InceptionV3	81.10	**86.67**	82.79	77.21	**89.25**	98	**40**	**260**	332

## Usage Notes

For convenience, the dataset can be downloaded in three image modalities for both the positive and negative COVID-19 parts:

- Images downloaded directly from XNAT, including the organization shown in Fig. 2, with images in DICOM format.
- MIDS format shown in Fig. 3.
- Preprocessed and stored in numpy array format to perform the experiments.

We encourage researchers to use this dataset to validate the performance of ML or DL models, aiming for true generalization in the detection of the disease. Although the results presented here when using CNNs for classification on the dataset are encouraging results, it is recommended to make more combinations and tests with it, being the decision of each researcher how to use it.

## Data Availability

The source code to generate the technical analysis has been uploaded to GitHub: https://github.com/BioAITeam/COVID19-Detection/tree/main/Cov-Caldas%20Dataset.
